# Megacystis-Microcolon-Intestinal Hypoperistalsis Syndrome Associated With Prune Belly Syndrome: A Case Report

**Published:** 2012-04-01

**Authors:** Tanveer Akhtar, Anand Alladi, OS Siddappa

**Affiliations:** Department of Paediatric Surgery, BMC and RI, Bangalore-560002. India

**Keywords:** Megacystis Microcolon Intestinal Hypoperistalsis Syndrome, Prune Belly Syndrome

## Abstract

Megacystis Microcolon Intestinal Hypoperistalsis Syndrome is a quite rare congenital anomaly that presents with a functional obstruction of the gastrointestinal tract which is usually fatal. It is three to four times more prevalent in females. We present a case of a rare association of a male neonate with Megacystis Microcolon Intestinal Hypoperistalsis Syndrome who in addition had the classical triad of Prune Belly Syndrome and thus suggest a possibility of different spectrums with a common pathogenesis.

## INTRODUCTION

Megacystis Microcolon Intestinal Hypoperistalsis Syndrome (MMIHS) is a rare autosomal recessive disorder that was first described in 1976 by Berdon et al., in five newborn girls [1]. It is characterized by abdominal distension, distended non-obstructive urinary bladder, microcolon, intestinal hypoperistalsis and malrotation of the small intestine. This entity was originally described as a pathology almost exclusively confined to females. Prune Belly Syndrome (PBS) on the other hand was classically a male entity, with the triad of a lax abdominal wall, bilateral cryptorchidism, and a dilated, dysmorphic urinary tract. We present an extremely rare association of a male neonate presenting to us with all the above criteria of MMIHS in addition to the classical triad of PBS and review the literature regarding the pathogenesis.

## CASE REPORT

A 2-day-old male neonate presented to us with a history of bilious vomiting and abdominal distension with very lax abdominal wall. There was no history of passage of meconium. Antenatal ultrasonography done in the third trimester showed dilated stomach and small bowel loops with distended bladder. Examination of the baby revealed a very lax abdominal wall with visible dilated bowel loops, visible and palpable bladder and kidneys (Fig 1). The child had bilateral impalpable testes, large pendulous penile shaft suggesting megalourethra and a normal anal opening. Erect X-ray abdomen showed grossly distended stomach, few air fluid levels and absence of gas distally. Contrast enema revealed microcolon with contrast filling the small bowel (Fig 2). On ultrasonography, there was bilateral gross hydroureteronephrosis, hugely distended and thick bladder and non visualization of both the testes. Exploratory laparotomy revealed grossly distended stomach, Ladd’s band obstructing the duodenum with duodenojejunal junction to the right of vertebra and caecum in the left hypochondrium confirming malrotation. There was microcolon with small bowel dilatation upto terminal ileum. Bladder was very thick and distended with bilateral gross hydroureteronephrosis. Both the testes were high intraabdominal in location (Fig 3). Ladd’s procedure was done. Considering a possibility of total colonic aganglionosis, terminal ileostomy with a mucous fistula was done. Appendectomy was done and a rectal biopsy taken just above the peritoneal reflection. Post operatively, the ileostomy failed to function and the child continued to have bilious aspirates and abdominal distension. Appendicular and rectal biopsy revealed normal ganglion cells. In view of poor outcome and long term complications, parents decided to discontinue the treatment on the seventh post operative day.

**Figure:**
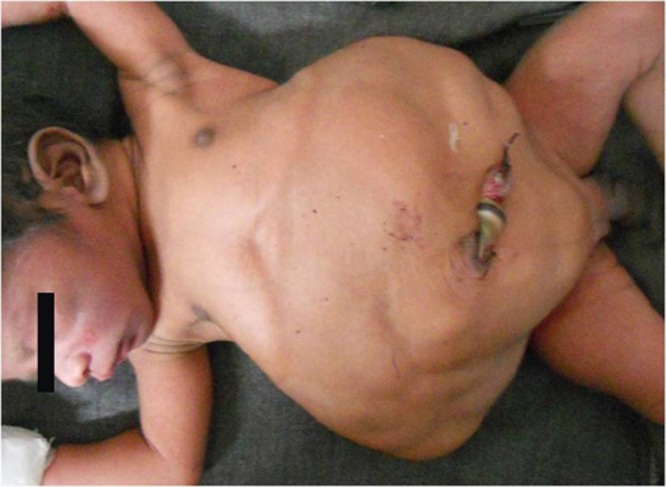
Figure 1: Prune belly

**Figure:**
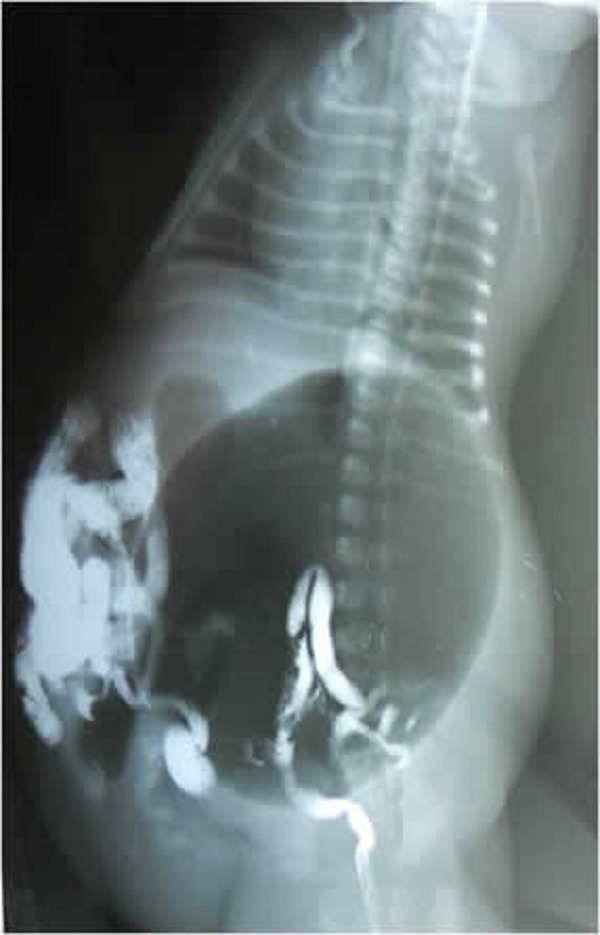
Figure 2: Contrast enema revealed microcolon with contrast filling the small bowel

**Figure:**
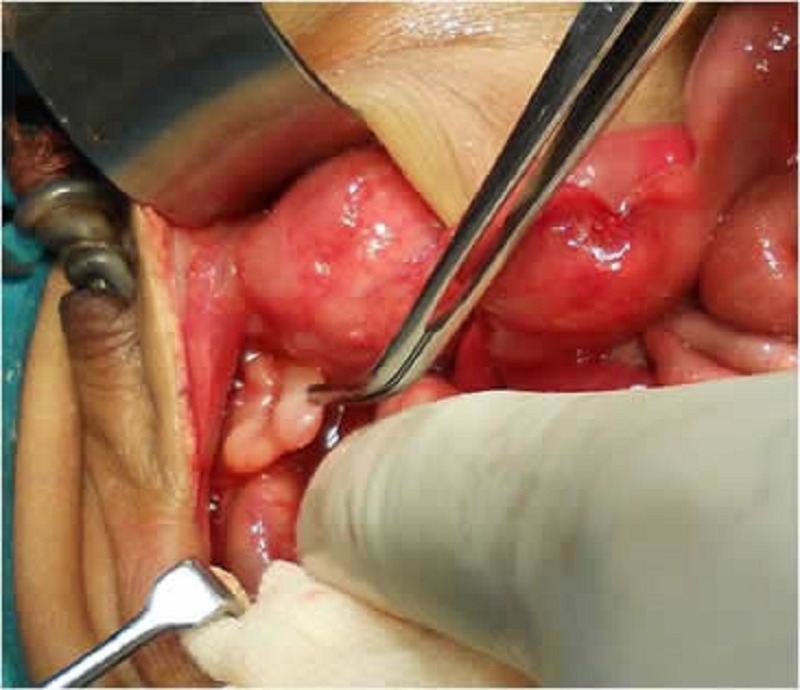
Figure 3: Intra-abdominal testes

## DISCUSSION

Till date, 227 cases of MMIHS have been reported in neonates. The frequent occurrence of the syndrome in infants of consanguineous parents and in siblings of affected child suggests an autosomal recessive mode of inheritance. Usually, infants present with bilious vomiting and abdominal distension caused by functional intestinal obstruction and bladder distension. Plain X-ray abdomen would reveal dilated bowel loops, huge gastric distension or a gasless abdomen. Contrast enema reveals a microcolon that is frequently malrotated [1-5].

Review of literature reveals a significant overlapping of clinical features in PBS and MMIHS, and the possibility of a common pathogenesis has been suggested [9]. Dilated urinary tract is classically present in both the syndromes. Lax abdominal wall is present in all classical cases of PBS which may be present in few cases of MMIHS. Cryptorchidism again is present in all classical cases of PBS which is infrequent in case of MMIHS [2,10] (Table 1). Anomalies of GIT have been reported in upto 30% of PBS cases, with malrotation and atresias accounting for majority. Imperforate anus and pouch colon have been described in association with PBS. It is the presence of intestinal hypoperistalsis and microcolon that distinguish these two syndromes [8,9]. Olivereira et al reported a case of PBS occurring in the brother of a female infant with MMIHS. The author suggested that the two syndromes reflected variations of the pathological spectrum originating from bladder distention during intrauterine life [9]. Levin et al reported the only other case of a male infant with classical triad of PBS as well as the radiographic and clinical features of MMIHS which was alike our case [10]. Chen et al described a female fetus with MMIHS who was found to have PBS on gross examination [11]. Histological studies of the myenteric and submucosal plexuses of the bowel of MMIHS patients have found normal ganglion cells in the majority of the patients, decreased in some, hyperganglionosis and giant ganglia in others.

**Figure:**
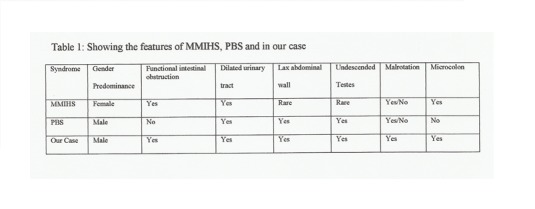
Table 1: Showing the features of MMIHS, PBS and in our case

An imbalance in intestinal peptides was suggested as one of the possible causes of hypoperistalsis. Absence of Interstitial Cell of Cajal (Pacemaker cells) in the bowel and urinary bladder has been reported as a causative factor. Puri and coworkers showed vacuolar degenerative changes in the smooth muscle cells with abundant connective tissue between muscle cells in the bowel and bladder. Several subsequent reports have confirmed evidence of intestinal myopathy in MMIHS. MMIHS has been reported with excessive smooth muscle glycogen storage postulating the pathogenesis involving a defect of glycogen-energy utilization. Other investigators have reported absence or marked reduction in smooth muscle actin and other contractile and cytoskeletal proteins in the smooth muscle layers of bowel in MMIHS [2]. PBS has been reported in association with Turner's syndrome, Monosomy 16, Trisomy 13 and 18, Perlman syndrome, Beckwith–Wiedemann syndrome, VACTERL association, Pfeiffer syndrome type 3, and MMIHS suggesting the possibility of a common pathogenesis [8].

Nutritional support is the mainstay of treatment. Palliative surgery such as jejunostomy or cystostomy is generally needed [6]. It has a poor prognosis with majority of reported infants dead. During the last few years, the improved neonatal total parenteral nutrition and success of bowel transplantation has increased the survival rates to nearly 20% (43 of 218 cases in whom outcome is reported) in children with MMIHS. Twelve multivisceral transplantations have been reported to date in patients with MMIHS [3,7].

In conclusion, we present a neonate that exhibited findings consistent with both MMIHS and PBS, and is the second case being reported here with both syndromes occurring concurrently. This suggests a possibility that these syndromes could have a common pathogenesis with different spectrums in clinical manifestations.

## Footnotes

**Source of Support:** Nil

**Conflict of Interest:** None declared

